# Short‐ and Long‐Term Effect of Multidomain Lifestyle Intervention on Frailty: Post Hoc Analysis of an RCT

**DOI:** 10.1111/jgs.19552

**Published:** 2025-05-30

**Authors:** Johanna Pöyhönen, Hanna‐Maria Roitto, Jenni Lehtisalo, Esko Levälahti, Timo Strandberg, Miia Kivipelto, Jenni Kulmala, Riitta Antikainen, Hilkka Soininen, Jaakko Tuomilehto, Tiina Laatikainen, Tiia Ngandu

**Affiliations:** ^1^ Department of Public Health Finnish Institute for Health and Welfare (THL) Helsinki Finland; ^2^ Department of Medicine Clinicum, University of Helsinki Helsinki Finland; ^3^ Department of Geriatrics Helsinki University Hospital Helsinki Finland; ^4^ Institute of Clinical Medicine/Neurology University of Eastern Finland Kuopio Finland; ^5^ Center for Life Course Health Research University of Oulu Oulu Finland; ^6^ Division of Clinical Geriatrics, Center for Alzheimer Research, Care Sciences and Society (NVS) Karolinska Institutet Stockholm Sweden; ^7^ Institute of Public Health and Clinical Nutrition University of Eastern Finland Kuopio Finland; ^8^ Ageing Epidemiology Research Unit (AGE), School of Public Health Imperial College London London United Kingdom of Great Britain and Northern Ireland; ^9^ Theme Inflammation and Aging Karolinska University Hospital Stockholm Sweden; ^10^ Faculty of Social Sciences (Health Sciences) and Gerontology Research Center (GEREC) Tampere University Tampere Finland; ^11^ Medical Research Center Oulu Oulu University Hospital Oulu Finland; ^12^ Neurocenter, Department of Neurology Kuopio University Hospital Kuopio Finland; ^13^ Department of International Health National School of Public Health Madrid Spain; ^14^ South Ostrobothnia Central Hospital Seinäjoki Finland; ^15^ Department of Public Health University of Helsinki Helsinki Finland; ^16^ North Karelia Wellbeing Service County (Siun Sote) Joensuu Finland

**Keywords:** frailty, multidomain lifestyle intervention, pre‐frailty, protein intake

## Abstract

**Background:**

The prevalence of frailty is increasing as the population ages. Lifestyle interventions have shown potential in frailty prevention. Intervention studies have been generally limited by short interventions and follow‐ups or by focusing on single‐domain approaches. We aimed to investigate whether a 2‐year multidomain lifestyle intervention prevents phenotypic pre‐frailty or frailty and whether baseline factors predict phenotypic pre‐frailty or frailty.

**Methods:**

A total of 1259 participants (aged 60–77 years) in the Finnish Geriatric Intervention Study to Prevent Cognitive Impairment and Disability (FINGER) were randomized to a multidomain intervention group or to a regular health advice group for 2 years. Frailty was defined by modified Fried phenotype. Pre‐frail and frail participants were grouped for analyses. The prevalence of pre‐frailty/frailty at baseline and at 2, 7, and 11 years, the change in prevalence from baseline, and the difference in these changes between intervention and control groups were estimated using a mixed‐effects logistic regression model.

**Results:**

The intervention reduced the risk of pre‐frailty/frailty up to 7 years. The prevalence decreased in the intervention group from baseline (47%) to 2 years (42%), while it increased in the control group (45% to 49%), resulting in a −9.6‐percentage point difference in the change (*p* = 0.007). After the active intervention period, the prevalence began to increase in both groups, but the difference in the change remained in favor of the intervention group at 7 years (−6.2 percentage points, *p* = 0.049). The beneficial effect was no longer evident at 11 years. Older age, lower protein intake, and a higher number of chronic diseases were strongly associated with pre‐frailty/frailty.

**Conclusions:**

A 2‐year multimodal lifestyle intervention effectively prevented phenotypic pre‐frailty/frailty, with sustained benefits observed up to 7 years. Continuous support for a healthy lifestyle may be necessary to prevent late‐life pre‐frailty or frailty.

## Graphical abstract

A Sankey diagram illustrating the flow of participants from baseline frailty status to each follow‐up assessment. Data represents numbers of participants observed in each group.

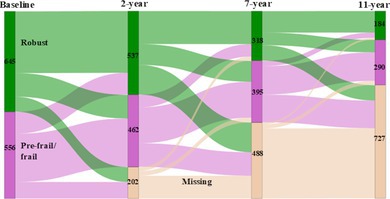

**Summary**

- Key points
  ○ A 2‐year multidomain lifestyle intervention reduced the risk of phenotypic pre‐frailty/frailty up to 7 years.
  ○ The effect was particularly prominent in the physical activity component of frailty.
  ○ Lower baseline protein intake predicted the development of phenotypic pre‐frailty/frailty up to 2 years.
- Why does this paper matter?
  ○ Healthcare providers are facing a growing number of older adults with frailty, and new knowledge on the prevention of frailty is essential. This is the first study demonstrating the effect of a multidomain lifestyle intervention with a long duration and follow‐up on phenotypic pre‐frailty and frailty.

## 1 Introduction

The prevalence of frailty is expected to increase due to population aging, making its prevention crucial. While there is limited evidence regarding whether frailty should be screened routinely, identifying and understanding frailty would be beneficial in clinical care [1]. Estimations of frailty prevalence vary depending on the definition used and the population studied [2, 3]. Frailty is commonly defined based on phenotype, which does not include diseases [4]. Other frequently used definitions include the frailty index (FI, accumulation of deficits) [5] and various frailty scales [6, 7].

It has been shown that different types of frailty, including definitions based on phenotype, accumulation of deficits scales, and frailty scores, can be reduced with single‐ and multidomain interventions [8, 9], although multidomain approaches may be more effective [9]. However, the intervention periods and follow‐ups in previous studies have generally been short. The interventions focusing on exercise have been shown to be effective in preventing or reducing phenotypic frailty [9, 10, 11, 12] and mobility disability [13]. One mechanism behind this might be the association between low physical activity, a component of phenotypic frailty, and chronic systemic inflammation and mitochondrial dysfunction [14, 15], which are also linked to the development of frailty [16, 17]. Eventually, the failure of multiple physiological systems leads to clinical manifestations and reduces resilience to stressors [1].

An increase in protein intake has also been shown to be effective in delaying and reducing frailty by various definitions including phenotype [18, 19]. Protein requirements are higher in older adults due to reduced muscle protein synthesis and increased loss of muscle mass [20]. In addition to their importance in muscle mass, strength, and function, specific amino acids may affect the pathophysiology of frailty by inhibiting inflammation [21].

Knowledge regarding the long‐term post‐intervention effect of a prolonged multidomain lifestyle intervention on phenotypic frailty is limited. A few studies with long intervention periods [22, 23] and post‐intervention follow‐ups [23] have shown reduced incidence of frailty [22] or lower FI [23], but these studies defined frailty using FI, which also includes diseases. Previously, we demonstrated a beneficial intervention effect on phenotypic pre‐frailty/frailty among men during the 2‐year intervention period of the Finnish Geriatric Intervention Study to Prevent Cognitive Impairment and Disability (FINGER) [24]. The aim of this study was to investigate whether this multidomain lifestyle intervention has a beneficial post‐intervention effect on physical pre‐frailty/frailty phenotype up to the 11‐year follow‐up. We also aimed to explore the role of baseline factors in the development of pre‐frailty and frailty during follow‐up.

## 2 Methods

### 2.1 Study Design and Participants

We used data from the FINGER, a multidomain randomized controlled lifestyle intervention study [25]. The active 2‐year intervention period was in 2009–2014, and the total follow‐up time was 11 years. The study recruited 1259 Finnish adults aged 60–77 years from the general population [26]. Participants were selected using the Cardiovascular Risk Factors, Aging, and Dementia (CAIDE) risk score of six points or higher [27] to indicate an elevated risk for dementia, and their cognitive performance was at or slightly below the expected mean level for their age according to the Finnish population. The recruitment occurred at six study centers in Finland.

### 2.2 Study Protocol

Participants were randomized to a multidomain intervention or control group (1:1). The intervention included four main components: nutrition, exercise, cognitive and social activity, and monitoring of metabolic and vascular risks. The control group received regular health advice.

Participants attended annual visits with a study nurse during the 2‐year intervention period, with follow‐up visits conducted at 5, 7, and 11 years. A physician performed a medical examination at screening and at 2 years, with a review of detailed medical history. The health questionnaire was completed at every visit, and physical performance (including the Short Physical Performance Battery (SPPB) and grip strength) was evaluated at baseline and at 2, 7, and 11 years. The structured Mini Nutritional Assessment (MNA) Short Form designed to screen for malnutrition [28, 29] was introduced at the 7‐year visit and collected also at the 11‐year visit. Relevant nutrient intakes were calculated from 3‐day food records at baseline and at 2 and 7 years (e.g., protein and energy intake). The detailed study protocol is described elsewhere [26].

The FINGER study was approved by the Coordinating Ethics Committee of the Hospital District of Helsinki and Uusimaa (94/13/03/00/2009 and HUS/1204/2017). The study was conducted according to the guidelines laid down in the Declaration of Helsinki, and written informed consent was obtained from all participants at the start of the study and at follow‐up visits. This study is registered with ClinicalTrials.gov (NCT01041989).

For the present study, we used the baseline data on frailty status, randomization group (intervention/control), age, sex, education (years), body mass index (BMI), number of chronic diseases (from medical history questionnaire filled out by a physician after the participant interview including high blood pressure, heart failure, angina pectoris, cancer, asthma, pulmonary emphysema or chronic bronchitis, angioplasty, coronary bypass, gallstones or gall bladder inflammation, rheumatoid arthritis, other articular disease, back illness, chronic urethritis or nephritis, cerebrovascular disease, diabetes, depression, and other psychological illnesses, other chronic disease), APOE genotype (dichotomized as Ɛ4 carrier vs. non‐carrier), annual household income (€), and protein intake (g/kg). Longitudinal data were considered for frailty status (2, 7, and 11 years), protein intake (2 and 7 years), and MNA (7 and 11 years).

### 2.3 Definition of Phenotypic Frailty

Phenotypic frailty was defined by the modified Fried criteria [4]. ***Weight loss*** was assessed with self‐reported loss of body weight during the previous 12 months, and ≥ 4.5 kg or ≥ 5% decrease were considered as weight loss. ***Weakness*** was assessed with hand‐grip strength (the maximum result from two measurements of each hand) measured with a hydraulic hand dynamometer applying the original cut‐offs by Fried (men BMI (kg/m^2^) ≤ 24: ≤ 29 kg, BMI 24.1–26: ≤ 30 kg, BMI 26.1–28: ≤ 30 kg, BMI > 28: ≤ 32 kg; women BMI ≤ 23: ≤ 17 kg, BMI 23.1–26: ≤ 17.3 kg, BMI 26.1–29: ≤ 18 kg, BMI > 29: ≤ 21 kg) [4]. ***Exhaustion*** was assessed with a question about feeling of weakness or tiredness during the previous month. Participants who reported “quite a lot” or “very much” were considered to have exhaustion. ***Low physical activity*** was assessed with a question asking: “How often do you in your leisure time exercise for at least 20 min so that you are at least mildly out of breath and sweaty?” Those who reported once a week or less or who had a disability or disease that does not allow exercise were considered to have low physical activity. ***Slowness*** was assessed with gait speed (the best result from two 4‐m walks at normal walking speed) with original Fried cut‐offs for 15 ft. applied for 4 m (men ≤ 173 cm: ≥ 6.15 s, > 173 cm: ≥ 5.26 s; women ≤ 159 cm: ≥ 6.15 s, > 159 cm: ≥ 5.26 s) [4].

Frailty status was classified as robust at 0 points, pre‐frail at 1–2 points, and frail at 3–5 points. Due to the limited number of frail individuals, frailty status was dichotomized (robust vs. pre‐frail/frail). If a participant scored one point on at least one of the frailty components, they were considered pre‐frail/frail even if data were missing on some components (baseline *n* = 48, 2 years *n* = 60, 7 years *n* = 113, 11 years *n* = 76). If the participant with missing components scored zero points on the non‐missing components, frailty status was considered missing (baseline *n* = 58, 2 years *n* = 89, 7 years *n* = 67, 11 years *n* = 60).

### 2.4 Statistical Analyses

Continuous variables at baseline are presented as means ± standard deviations and categorical variables as frequencies (%). Group comparisons were performed with Student's *t* test, Mann–Whitney U test, or Chi‐square test as appropriate.

Protein intake at baseline was used as a continuous variable in the analyses, but for the purpose of the prevalence comparison with frailty status it was dichotomized based on the recommendation of 1.2 g/kg (above/below) [30]. MNA was categorized into three classes: good nutrition (12–14 points), at risk of malnutrition (8–11 points), and malnutrition (≤ 7 points).

A mixed‐effects logistic regression model was used to analyze the effect of the intervention on frailty status (robust and pre‐frail/frail) at 2‐, 7‐, and 11‐year follow‐ups. The best model was identified using Akaike's information criterion (AIC) as a non‐linear model, where time was split into two linear variables (t2 = 0–2 years and t11 = 2–11 years). The analyses were adjusted for age, sex, years of education, baseline number of chronic diseases (0, 1, 2, or ≥ 3), and baseline protein intake (g/kg). To exploit all available data on frailty status, we applied imputation with mean values in adjusting covariates (baseline protein intake, diseases, and education years; maximum imputations *n* = 10). The interaction between randomization group and time was investigated. Interactions between each adjusting covariate and time were additionally investigated, and significant interactions (randomization group × t2/t11, baseline protein intake × t2/t11 and age × t2/t11) were retained in the model. Each frailty component (yes/no) was analyzed with a simple model without additional interactions. A sensitivity analysis was conducted similarly using the outcome of pre‐frailty/frailty defined by 2–5 frailty points. Additionally, we stratified the analyses by baseline frailty status.

Marginal mean probabilities were used to estimate prevalences of pre‐frailty/frailty in the study population at baseline and at 2, 7, and 11 years. The change in prevalence from baseline to each time‐point, and the differences in the change between intervention and control groups, as well as between different baseline ages and protein intakes, were tested by estimating specific combinations of marginal mean probabilities. The same analyses were conducted for individual frailty components. From the baseline frailty status stratified model, we investigated transition probabilities (from baseline to each time point) from robust to pre‐frail/frail and vice versa.

P‐value of < 0.05 was considered significant, and results are reported with 95% confidence intervals. Statistical analyses were performed using SPSS 29.0 for Windows (SPSS Inc., Chicago, IL, USA) and STATA 17.0 software. RStudio (version 2024.04.1, R Foundation, Vienna, Austria) was used to create the Sankey diagram. Mixed‐effects logistic regression models were estimated using the melogit STATA program with the mean–variance adaptive Gauss–Hermite quadrature integration method. In case of estimation convergence issues for gait speed outcome, likely caused by small group sizes, the non‐adaptive Gauss–Hermite quadrature integration method was used.

## 3 Results

Of the 1259 participants in the FINGER study, 1201 (95%) with baseline frailty status data available were included in the analyses (**Figure** S1). At baseline, the mean age of participants was 69.3 years, and 551 (46%) were women. Of all participants, 645 (54%) were robust, 541 (45%) pre‐frail, and 15 (1%) frail. In the analyses, pre‐frail and frail groups were combined (*n* = 556, 46%). Participants who were pre‐frail/frail had a higher BMI, more chronic diseases, lower protein intake, slower gait speed, and weaker grip strength than those in the robust group (Table 1). Robust participants in the control group were younger than those in the intervention group (*p* = 0.042). No other differences in baseline frailty subgroups were found between intervention and control groups.

**TABLE 1:** Baseline characteristics of participants; comparison between robust and prefrail/frail groups.

Characteristics	N	All participants (*n* = 1201)	Robust (*n* = 645, 53.7%)	Pre‐frail or frail (*n* = 556, 46.3%)	*P*‐value
Intervention group	1201	603 (50.2)	318 (49.3)	285 (51.3)	0.499
Sociodemographics and ‐economics					
Age (years)	1201	69.3 ± 4.7	69.3 ± 4.6	69.4 ± 4.7	0.816
Female sex	1201	551 (45.9)	283 (43.9)	268 (48.2)	0.134
Education (years)	1200	10.0 ± 3.4	10.1 ± 3.5	9.9 ± 3.3	0.315
Annual household income, €	1149				0.392
0–20,000		263 (22.9)	130 (20.8)	133 (25.3)	
20,001–30,000		305 (26.5)	165 (26.4)	140 (26.7)	
30,001–40,000		241 (21.0)	134 (21.5)	107 (20.4)	
40,001–50,000		132 (11.5)	74 (11.9)	58 (11.0)	
Over 50,000		208 (18.1)	121 (19.4)	87 (16.6)	
Health factors					
Body mass index (kg/m^2^)	1197	28.2 ± 4.7	27.5 ± 4.2	29.0 ± 5.2	< 0.001
Diseases^a^	1195	2.5 ± 1.5	2.3 ± 1.5	2.7 ± 1.5	< 0.001
None		111 (9.3)	70 (10.9)	41 (7.4)	
1		226 (18.9)	141 (22.0)	85 (15.3)	
2		312 (26.1)	176 (27.5)	136 (24.5)	
≥ 3		546 (45.7)	253 (39.5)	293 (52.8)	
APOE Ɛ4 carrier (yes)^b^	1119	363 (32.4)	200 (33.1)	163 (31.7)	0.603
Protein intake (g/kg)	1191	0.99 ± 0.33	1.02 ± 0.32	0.96 ± 0.34	< 0.001
Frailty components^c^					
1. Weight loss (yes)	1198	106 (8.8)	NA	106 (19.2)	NA
2. Weakness: grip strength (yes)	1170	124 (10.6)	NA	124 (23.6)	NA
3. Exhaustion (yes)	1190	79 (6.6)	NA	79 (14.5)	NA
4. Low physical activity (yes)	1197	364 (30.4)	NA	364 (65.9)	NA
5. Slowness: gait speed (yes)	1172	23 (2.0)	NA	23 (4.4)	NA
Frailty points total	1201	0.6 ± 0.7	NA	1.3 ± 0.5	NA
Grip strength (kg)	1174	34.4 ± 10.8	36.1 ± 9.9	32.5 ± 11.5	< 0.001
Gait speed (s)	1176	3.43 ± 0.87	3.26 ± 0.56	3.63 ± 1.10	< 0.001

*Note:* Data are numbers (%) of participants or means ± SD. Comparison analyzed with Chi‐square or non‐parametric test.

Abbreviations: APOE, Apolipoprotein E; NA, not applicable.

^a^ Mean number of 18 diagnoses (high blood pressure, heart failure, angina pectoris, cancer, asthma, pulmonary emphysema or chronic bronchitis, angioplasty, coronary bypass, gallstones or gall bladder inflammation, rheumatoid arthritis, other articular disease, back illness, chronic urethritis or nephritis, cerebrovascular disease, diabetes, depression, and other psychological illnesses, other chronic disease); self‐reported at baseline if a physician had diagnosed or treated the condition during the previous 12 months.

^b^ Carrier of at least one APOE Ɛ4 allele vs. non‐carrier.

^c^ Frailty components: 0/1, *n* (%) of participants scoring a point; point scoring explained in text; frailty total points: mean points (5 max).

**FIGURE 1:**
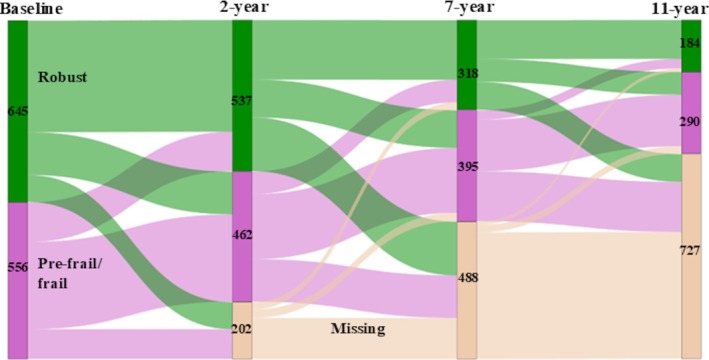
A Sankey diagram illustrating the flow of participants from baseline frailty status to each follow‐up assessment. Missing frailty status data after baseline are also shown. The diagram shows a decrease in the robust group and an increase in the pre‐frail/frail group. The missing data group was the largest at the final follow‐up. Data represent numbers of participants observed in each group.

At the 11‐year follow‐up, 474 participants (39%) had data on frailty status (184/15% robust, 264/22% pre‐frail, 26/2% frail) and 727 participants (61%) had missing data. The observed prevalence and transitions of participants between frailty subgroups and the missing group are shown in Figure 1. When comparing the group with missing frailty status data with the group with available data at 11 years, the participants in the missing group were older, had more chronic diseases, a higher BMI, lower education, lower protein intake, weaker grip strength, and slower gait speed at baseline. However, no difference emerged in randomization group between participants with missing frailty status data at 11 years (*p* = 0.907), nor was a difference seen in their baseline frailty status (*p* = 0.318).

**FIGURE 2:**
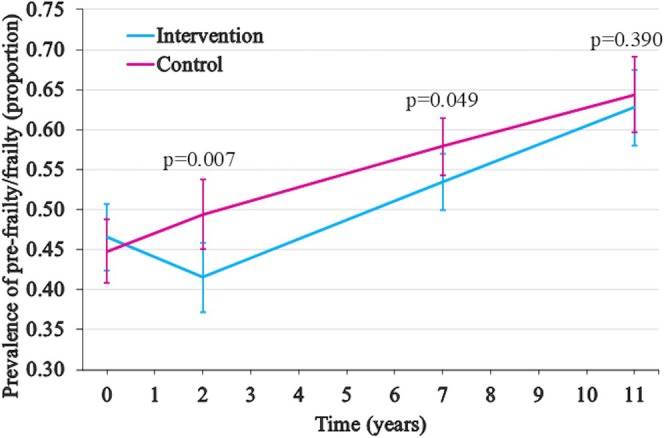
A graph of estimated mean prevalences (range of proportions 0.00–1.00) of pre‐frail/frail participants in randomization groups. The estimates are from the best‐fitting adjusted mixed‐effects logistic regression model, where time was non‐linear, split into two linear periods of 0–2 years and 2–11 years, including an interaction between randomization group (intervention vs. control) and time. P‐values indicate the difference in the prevalence change between the intervention and control groups from baseline to each follow‐up. Error bars depict 95% confidence intervals.

The intervention reduced the risk of pre‐frailty/frailty up to 7 years (Figure 2). The difference in the estimated change from baseline to 2 years was −9.6 percentage points (95% CI −16.6—(−2.6), *p* = 0.007) (Table 2). The prevalence decreased in the intervention group, while it increased in the control group. After the 2‐year intervention period, the prevalence increased linearly in both groups, but the increase from baseline remained smaller in the intervention group up to 7 years. The difference in the estimated change between randomization groups from baseline to 7 years was −6.2 percentage points (95% CI −12.3—0.0, *p* = 0.049). This beneficial intervention effect was attenuated at 11 years. Sensitivity analysis with the outcome of pre‐frailty/frailty defined by 2–5 frailty points showed a preventive effect up to 2 years, with a difference between randomization groups of −4.3 percentage points (95% CI −8.5 – (−0.1), *p* = 0.045). At the 7‐year follow‐up, the preventive effect remained close to statistical significance (*p* = 0.080, Table S1). When stratifying the analyses by baseline frailty status, the intervention resulted in the prevention of new cases of pre‐frailty/frailty among robust participants for 2 years and reversion among pre‐frail/frail participants in the long term (Table S2).

**TABLE 2:** Estimated prevalence of pre‐frailty/frailty at baseline and at 2, 7, and 11 years of follow‐up, the estimated change in prevalence from baseline, and the difference in estimated change between intervention and control groups.

Year	Group	Estimated prevalence (95% CI)	Estimated change from baseline (95% CI)	*p*	Difference in estimated change between intervention and control groups (95% CI)	*p*
0	I	46.6 (42.5–50.6)	NA	NA	NA	NA
0	C	44.8 (40.8–48.9)	NA	NA	NA	
2	I	41.5 (37.3–45.7)	−5.0 (−10.0–(−0.1))	0.044	−9.6 (−16.6–(−2.6))	0.007
2	C	49.4 (45.1–53.7)	4.6 (−0.4–9.6)	0.070		
7	I	53.4 (49.9–57.0)	6.9 (2.5–11.2)	0.002	−6.2 (−12.3–0.0)	0.049
7	C	57.9 (54.3–61.4)	13.0 (8.7–17.4)	< 0.001		
11	I	62.8 (58.1–67.4)	16.2 (10.9–21.5)	< 0.001	−3.3 (−10.9–4.3)	0.390
11	C	64.4 (59.6–69.1)	19.5 (14.1–24.9)	< 0.001		

*Note:* The best‐fitting mixed‐effects logistic regression model, where time had a non‐linear effect and was split into two linear periods (t2 = 0–2 years, t11 = 2–11 years), was used to estimate the prevalence of pre‐frailty/frailty (%), the change from baseline (percentage points), and the difference in this change (percentage points) between intervention and control groups (time x randomization group interaction). Baseline age, sex, protein intake (g/kg), number of chronic diseases (0, 1, 2, ≥ 3), and education (years) were used as covariates. In the difference between groups, a negative value indicates that the difference is in favor of the intervention group.

Abbreviations: C, control; CI, confidence interval; I, intervention.

Older age (*p* = 0.002), lower baseline protein intake (*p* < 0.001), and a higher number of chronic diseases at baseline (*p* < 0.001) were all strongly associated with pre‐frailty/frailty during the study (Table 3). Female sex approached statistical significance (*p* = 0.061). We further analyzed interactions between time and the covariates (i.e., prediction of developing pre‐frailty/frailty over 11 years). Lower protein intake predicted the development of pre‐frailty/frailty up to 2 years (*p* = 0.005). While this effect weakened, it remained close to statistical significance at 7 years (*p* = 0.058). Older age predicted the development of pre‐frailty/frailty up to 11 years (at 2‐year follow‐up, *p* = 0.046; at 7‐ and 11‐year follow‐ups, *p* < 0.001). No other interactions with time were observed.

**TABLE 3:** Odds ratios of baseline factors associated with frailty from the adjusted mixed‐effects logistic regression model predicting the development of pre‐frailty/frailty during the 11‐year follow‐up.

	OR (95% CI)		*p*
	Robust	Pre‐frail/frail	
Age (years)	1	1.05 (1.02–1.08)	0.002
Sex (female)	1	1.29 (0.99–1.68)	0.061
Education (years)	1	0.99 (0.96–1.03)	0.765
Diseases (category)^a^	1	1.39 (1.22–1.59)	< 0.001
Baseline protein intake (g/kg)	1	0.36 (0.24–0.55)	< 0.001

*Note:* Odds ratios from the best‐fitting adjusted mixed‐effects logistic regression model, where time had a non‐linear effect and was split into two linear periods (t2 = 0–2 years, t11 = 2–11 years), including an interaction between randomization group and time.

Abbreviations: CI, confidence interval; OR, odds ratio.

^a^ Count of 18 diagnoses; self‐reported at baseline if a physician had diagnosed or treated the condition during the previous 12 months. Categories: 0, 1, 2, and ≥ 3.

We also investigated cross‐sectionally how protein intake under the recommended level (1.2 g/kg) at baseline and at 2 and 7 years (no data at 11 years) and malnutrition (MNA) at 7 and 11 years (no data at baseline or at 2 years) were associated with frailty status. The prevalence of protein intake under the recommended level was high at baseline and at 2 and 7 years. At 7 and 11 years, pre‐frail/frail individuals had more often insufficient protein intake and were more often at risk of malnutrition than robust individuals (Table S3).

We performed the same analyses for individual frailty components (Table S4). The intervention reduced the risk of low physical activity up to 7 years. The difference in the estimated change between groups at 2 years was −9.7 percentage points (95% CI −15.4 – (−4.1), *p* = 0.001). The prevalence of low physical activity decreased during the active intervention in the intervention group but increased in the control group. After 2 years, the prevalence increased also in the intervention group, but the change from baseline was still smaller in the intervention group than in the control group at 7 years (the group difference − 7.1 percentage points, 95% CI −12.3 – (−1.9), *p* = 0.007). The beneficial effect was attenuated at 11 years. No effects of the intervention were found on any of the other individual frailty components.

## 4 Discussion

We found that the 2‐year multidomain lifestyle intervention prevented phenotypic pre‐frailty/frailty up to 7 years. The effect was both prevention of new cases among robust participants and reversion of pre‐frailty/frailty to robustness. The decrease in prevalence was evident in the intervention group up to 2 years and started to increase in both groups after the active intervention period. The difference between groups remained at 7 years but no longer at 11 years. The intervention effect was particularly pronounced in physical activity. Lower baseline protein intake, older age, and a higher number of chronic diseases were associated with pre‐frailty/frailty. Older age (for up to 11 years) and lower baseline protein intake (for up to 2 years) predicted the development of pre‐frailty/frailty. The mid‐ and long‐term follow‐ups showed strong overlap between protein intake under the recommended level, risk of malnutrition/malnutrition, and pre‐frailty/frailty.

To our knowledge, only a few studies with a similar long multidomain intervention and an extended post‐intervention follow‐up as in our study exist. In a study of ≥ 70‐year‐old community‐dwelling adults, a 3‐year multidomain intervention (diet, physical activity advice, and cognitive training) had a beneficial effect on the incidence but not the severity of frailty defined by FI [22]. Compared with our study, participants in that study were older, had more frailty at baseline, and the baseline mean FI was in the pre‐frailty range in both the intervention and control groups, as frailty (FI ≥ 0.25) was the outcome measure. Also, that study had no post‐intervention follow‐up. The physical activity component of the intervention was solely advice, and as the authors concluded, providing actual physical exercise might enhance the effect on the severity of frailty. Another study with a 10‐year multidomain intervention (dietary advice with caloric restriction and increased physical activity) showed a lower FI for the intervention group even 8 years post‐intervention [23]. However, the participants were younger (45–71 years) and had type 2 diabetes and obesity or overweight, differing from our study population. Also in that study, the mean FI was within the range of pre‐frailty at baseline. Interestingly, in contrast to our study, that trial did not show an association between the 10‐year lifestyle intervention and later phenotypic frailty (without weight loss component), but the study had no frailty assessment at baseline, i.e., the change could not be evaluated [31].

A review with 14 trials highlighted that frailty (by different definitions) can be prevented or reduced with different intervention combinations of different lengths (e.g., exercise; exercise and dietary advice; exercise, diet and cognitive training) [8]. However, all these interventions were conducted in pre‐frail or frail participants, and interventions and follow‐ups were mainly short. Also, the authors pondered that the interventions should address various aspects of frailty. Our study supports this preventive effect while also providing additional insights into the long‐term effect of a prolonged intervention, using robust participants as a reference. In addition to physical frailty, multidomain lifestyle intervention may potentially be beneficial for other aspects of frailty (i.e., social and psychological frailty).

Our findings indicate that low physical activity was the component that was the most improved by the intervention up to 7 years, supporting results from earlier studies that exercise has been effective in the prevention of frailty by various definitions [9, 10]. The previous analysis of FINGER data showed that the intervention may reverse phenotypic frailty during 2 years, particularly among physically inactive men [24]. The differences in the definitions of weight loss and physical activity as well as varying approaches in data analyses may explain the discrepancies in our results at 2 years compared with earlier results.

Supporting previous studies, we found that protein intake could be an important target for intervention. Protein intake requirements are higher in older adults, particularly to gain benefits of exercise interventions due to anabolic resistance (e.g., decreased muscle uptake of dietary amino acids) [30]. Also, specific amino acids along with exercise may affect the pathophysiology of frailty by inhibiting inflammation [14, 21]. A review scoring intervention effects and implementations found that strength training and protein supplementation are the most effective in delaying or reversing frailty (by different definitions) and also are the easiest to implement in primary care [18]. An observational study in women reported a dose‐dependent association between higher protein consumption and a lower incidence of phenotypic frailty [19]. A recent review summarizing the evidence from meta‐analyses and systematic reviews of randomized controlled trials suggested that community‐dwelling older adults and hospitalized older adults with frailty may benefit from oral nutritional supplementation [1].

There are some limitations in this study. First, comparisons with the previous above‐mentioned studies should be handled with caution due to different definitions of frailty. We used a phenotypic definition of frailty that has a clear pre‐defined set of criteria, and we have previously shown a beneficial short‐term effect on this outcome. Throughout the 11 years, the number of frail participants remained low in the pre‐frail/frail group (the maximum was 5.5% at 11 years among all participants with data on frailty status), making it difficult to draw conclusions about preventing frailty alone. However, results from sensitivity analyses provided some evidence that the preventive effect is consistent even with more severe pre‐frailty (2–5 frailty points) during the active intervention period. The phenotypic frailty definition was aligned with the original Fried phenotype as much as possible [4]. Weight loss was originally classified as involuntary, but that information was not available in the current study and was self‐reported. Physical activity assessment was done with a different method (originally calculated as kcal expended). Also, participants were classified as pre‐frail/frail if at least one component was present, even with missing data on some components, and as missing if none of the components were met and some missing values. The number of participants with missing data components stayed low throughout the trial. Additionally, the original study was designed with a different primary outcome. Some group sizes, especially in the individual phenotypic frailty component analyses, are small, and thus, the results should be interpreted with caution due to limited statistical power. We also need to consider the growing proportion of missing data at each follow‐up. The mixed‐effect logistic regression analysis assumes the missing data is random. There was no difference in the randomization group among the participants with missing data at 11 years. However, it is possible that more participants who dropped out were frail than those who remained, suggesting the possibility of survival bias. Strengths of this study, in turn, include a carefully planned design, randomization, a long intervention period, extended follow‐up, and a large study population. The recruited participants were at risk for dementia and, as frailty shares many risk factors with dementia [17], the FINGER study provided a valuable opportunity to study pre‐frailty and frailty.

Frailty status has been shown to be dynamic [32], offering an opportunity for prevention or reduction of this geriatric syndrome. We have previously demonstrated that people with pre‐frailty may particularly benefit from intervention in relation to cognition [33]. The risks of adverse health outcomes have consistently been shown to be greater among frail individuals. In this light, our results on potential preventive interventions are encouraging. Multidomain lifestyle interventions with continuous support should be implemented into healthcare for older adults at risk of dementia to improve individual health outcomes and to decrease the frailty‐related burden on healthcare systems.

## 5 Conclusions

The 2‐year multimodal lifestyle intervention prevented phenotypic pre‐frailty/frailty during the intervention period, and the preventive effect persisted up to 7 years but was no longer evident at 11 years. The effect was primarily driven by physical activity. Continuous support for a healthy lifestyle in people at risk of dementia may be necessary to prevent late‐life pre‐frailty or frailty. Lower protein intake is a strong independent short‐term predictor of phenotypic pre‐frailty/frailty. Additionally, late‐life pre‐frailty/frailty strongly overlaps with the risk of malnutrition and insufficient protein intake, yielding potential targets for prevention.

## Author Contributions

Tiia Ngandu, Riitta Antikainen, Tiina Laatikainen, Timo Strandberg, Jaakko Tuomilehto, Hilkka Soininen, and Miia Kivipelto planned and designed the study. Tiia Ngandu, Jenni Lehtisalo, and Miia Kivipelto coordinated the study. Johanna Pöyhönen carried out the statistical analyses with the help of Esko Levälahti. Johanna Pöyhönen, Jenni Lehtisalo, Hanna‐Maria Roitto, and Tiia Ngandu interpreted the results and together with Jenni Kulmala drafted the report. All authors have read and approved the final manuscript.

## Disclosure

Financial sponsors played no role in study design, methods, subject recruitment, collection, analysis, and interpretation of data, or preparation of the paper.

## Conflicts of Interest

The authors declare no conflicts of interest.

## Supporting information


Data S1.

## Data Availability

The data presented in this article are not available because public deposition of the de‐identified data is not possible for legal and ethical reasons, and complete de‐identification is not possible as this study is part of an ongoing study. Participants gave informed consent, which includes data use only under a confidentiality agreement. The data contain sensitive information, and public data deposition may cause privacy concerns. Those fulfilling the requirements for viewing confidential data in accordance with Finnish law and the Finnish Institute for Health and Welfare are able to access the data after completion of the material transfer agreement. Requests to access the data should be directed to kirjaamo@thl.fi.
