# Supplementary material for: Short‐ and Long‐Term Effect of Multidomain Lifestyle Intervention on Frailty: Post Hoc Analysis of an RCT
Source: J Am Geriatr Soc. 2025 May 30;73(8):2457–65. doi: 10.1111/jgs.19552 (PMC12396167; doi:10.1111/jgs.19552)
Supplement: Supplementary file 1 — Data S1. [file JGS-73-2457-s001.pdf]

## Supplementary material

**Figure S1. Flow chart.** A total of 1201 of 1259 participants in the FINGER trial with baseline data on frailty (modified intention to treat) were selected for the present study.

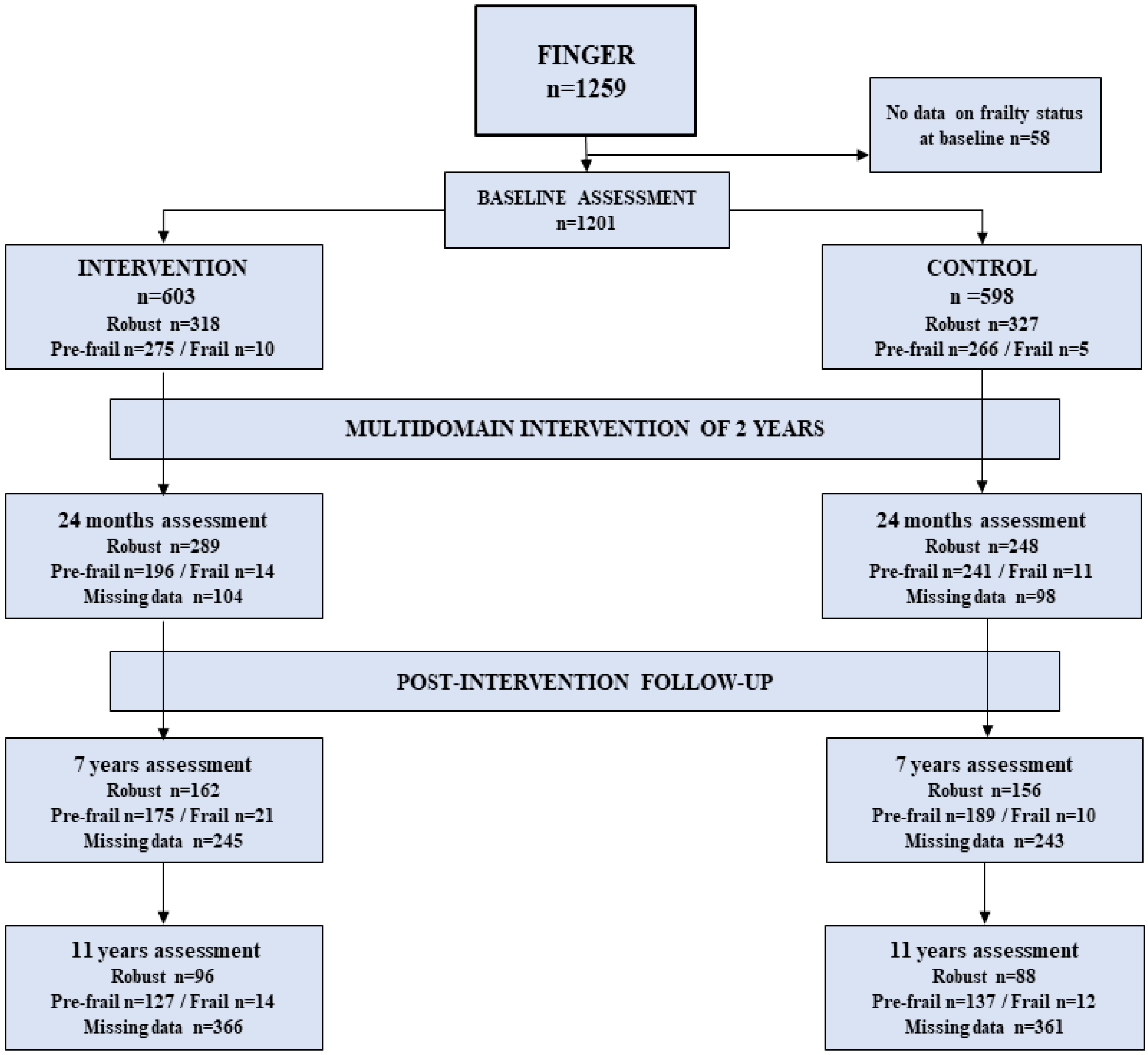

**Table S1**. Estimated prevalence of pre-frailty/frailty (2–5 frailty points) at baseline, 2, 7 and 11 years of follow-up, the estimated change in prevalence from baseline, and the difference in estimated change between intervention and control groups

| Year | Group | Estimated prevalence (95% CI) | Estimated change from baseline (95% CI) | *P*-value | Difference in estimated change between intervention and control groups (95% CI) | *P*-value |
|---|---|---|---|---|---|---|
| 0 | I | 9.1 (6.8–11.3) | NA | NA | NA | NA |
| 0 | C | 8.1 (6.0–10.2) | NA | NA | NA | |
| 2 | I | 9.3 (6.9–11.7) | 0.2 (-2.6–3.1) | 0.864 | -4.3 (-8.5–(-0.1)) | 0.045 |
| 2 | C | 12.6 (9.8–15.4) | 4.5 (1.5–7.6) | 0.004 | | |
| 7 | I | 15.5 (12.9–18.0) | 6.4 (3.6–9.2) | <0.001 | -3.6 (-7.7–0.4) | 0.080 |
| 7 | C | 18.2 (15.4–20.9) | 10.1 (7.1–13.0) | <0.001 | | |
| 11 | I | 22.1 (17.8–26.3) | 13.0 (8.6–17.4) | <0.001 | -2.4 (-8.6–3.8) | 0.443 |
| 11 | C | 23.5 (19.2–27.9) | 15.4 (11.0–19.9) | <0.001 | | |

The best-fitting mixed-effects logistic regression model, where time had a non-linear effect and was split into two linear periods (t2=0–2 years, t11=2–11 years), was used to estimate the prevalence of pre-frailty/frailty with 2–5 frailty points (%), the change from baseline (percentage points), and the difference in this change (percentage points) between intervention and control groups (time x randomization group interaction). Baseline age, sex, protein intake (g/kg), number of chronic diseases (0, 1, 2, ≥3), and education (years) were used as covariates. In the difference between groups, a negative value indicates that the difference is in favor of the intervention group. *Abbreviations: CI=confidence interval, I=intervention, C=control.*

**Table S2**. Transition probabilities (%) from baseline robust group to pre-frail/frail group and vice versa at 2, 7 and 11 years

| | **Baseline frailty status** | | | |
|---|---|---|---|---|
| | **Robust** | ***P*-value** | **Pre-frail/frail** | ***P*-value** |
| **2 years frailty status** | pre-frail/frail | | robust | |
| **I** | 19.9 | | 36.3 | |
| **C** | 31.6 | | 25.6 | |
| **Group difference (CI95%)** | -11.7 (-20.8–(-2.6)) | 0.012 | 10.8 (-0.2–21.7) | 0.054 |
| **7 years frailty status** | pre-frail/frail | | robust | |
| **I** | 39.2 | | 33.3 | |
| **C** | 41.3 | | 22.4 | |
| **Group difference (CI95%)** | -2.2 (-9.4–5.1) | 0.560 | 10.9 (3.6–18.2) | 0.004 |
| **11 years frailty status** | pre-frail/frail | | robust | |
| **I** | 57.4 | | 30.9 | |
| **C** | 49.6 | | 20.0 | |
| **Group difference (CI95%)** | 7.9 (-2.2–17.9) | 0.124 | 10.9 (1.5–20.3) | 0.022 |

The best-fitting mixed effects logistic regression model, where time had a non-linear effect and was split into two linear periods (t2=0–2 years, t11=2–11 years), was used to estimate transition probabilites (%) and difference between randomization groups (percentage points) from baseline robust group to pre-frail/frail group and vice versa at 2, 7 and 11 years. The model was adjusted with baseline age, sex, number of chronic diseases (categories 0, 1, 2 ≥3), education (years), and protein intake (g/kg).
*Abbreviations: I=intervention, C=control, CI=confidence interval*.

**Table S3**. Observed proportions of participants with protein intake under recommendation at baseline and at 2- and 7-year follow-up. Observed malnutrition prevalences in frailty subgroups at 7- and 11-year follow-up

| | Protein intake under recommendation (g/kg)[a] | | | MNA[b] | | | |
|---|---|---|---|---|---|---|---|
| | No | Yes | *P*-value[c] | good nutrition | at risk of malnutrition | malnutrition | *P*-value[c] |
| Baseline | | | | | | | |
| All participants n=1191 | 277 (23.3) | 914 (76.7) | | NA | NA | NA | |
| Robust | 158 (24.6) | 484 (75.4) | 0.232 | NA | NA | NA | NA |
| Pre-frail/frail | 119 (21.7) | 430 (78.3) | | NA | NA | NA | |
| 2 years | | | | | | | |
| All participants n=964 | 218 (22.6) | 746 (77.4) | | NA | NA | NA | |
| Robust | 129 (24.6) | 396 (75.4) | 0.112 | NA | NA | NA | NA |
| Pre-frail/frail | 89 (20.3) | 350 (79.7) | | NA | NA | NA | |
| 7 years | | | | | | | |
| All participants n=515, 668 | 95 (18.4) | 420 (81.6) | | 553 (82.8) | 105 (15.7) | 10 (1.5) | |
| Robust | 63 (24.0) | 199 (76.0) | <0.001 | 276 (90.2) | 29 (9.5) | 1 (0.3) | <0.001 |
| Pre-frail/frail | 32 (12.6) | 221 (87.4) | | 277 (76.5) | 76 (21.0) | 9 (2.5) | |
| 11 years | | | | | | | |
| All participants n=448 | NA | NA | | 398 (88.8) | 47 (10.5) | 3 (0.7) | |
| Robust | NA | NA | NA | 172 (95.0) | 8 (4.4) | 1 (0.6) | 0.002 |
| Pre-frail/frail | NA | NA | | 226 (84.6) | 39 (14.6) | 2 (0.7) | |

All participants with protein intake data at baseline, 2-, and 7-year follow-up and MNA data at 7- or 11-year follow-up out of total 1201 participants with baseline frailty status data who were accepted in the study analyses. MNA was added to the trial at 7 years, protein intake was not collected at 11 years. Data are numbers (%).

[a]Protein intake yes/no under the recommendation of 1.2 g/kg.

[b]MNA categorized into good nutrition (12–14 points), at risk of malnutrition (8–11 points), malnutrition (≤7 points).

[c]Chi-square test

*Abbreviations: MNA= Mini Nutritional Assessment. NA= Not applicable*

**Table S4**. Estimated prevalence of each frailty component at baseline and at 2,7 and 11 years of follow-up, the estimated change in prevalence from baseline, and the difference in estimated change between intervention and control groups

| | Year | Group | Estimated prevalence (95% CI) | Estimated change from baseline (95% CI) | P-value | Difference in estimated change between intervention and control groups (95% CI) | P-value |
|---|---|---|---|---|---|---|---|
| Physical activity | 0 | I | 29.7 (26.3–33.2) | NA | NA | NA | NA |
| | 0 | C | 27.8 (24.3–31.2) | NA | NA | NA | NA |
| | 2 | I | 23.3 (19.9–26.7) | -6.5 (-10.4–(-2.5)) | 0.001 | -9.7 (-15.4–(-4.1)) | 0.001 |
| | 2 | C | 31.1 (27.4–34.7) | 3.3 (-0.8–7.4) | 0.114 | | |
| | 7 | I | 32.2 (29.1–35.4) | 2.5 (-1.1–6.1) | 0.170 | -7.1 (-12.3–(-1.9)) | 0.007 |
| | 7 | C | 37.4 (34.0–40.8) | 9.6 (5.9–13.3) | <0.001 | | |
| | 11 | I | 40.4 (35.8–45.0) | 10.7 (5.9–15.5) | <0.001 | -4.3 (-11.2–2.6) | 0.218 |
| | 11 | C | 42.8 (38.0–47.6) | 15.0 (10.1–20.0) | <0.001 | | |
| Exhaustion | 0 | I | 5.4 (3.6–7.1) | NA | NA | NA | NA |
| | 0 | C | 4.9 (3.2–6.5) | NA | NA | NA | NA |
| | 2 | I | 5.9 (4.0–7.9) | 0.6 (-1.6–2.7) | 0.603 | 0.3 (-2.6–3.2) | 0.832 |
| | 2 | C | 5.1 (3.4–6.9) | 0.2 (-1.8–2.3) | 0.810 | | |
| | 7 | I | 7.0 (5.2–8.8) | 1.6 (-0.3–3.5) | 0.100 | -0.2 (-2.9–2.5) | 0.868 |
| | 7 | C | 6.7 (5.0–8.5) | 1.8 (0.0–3.7) | 0.056 | | |
| | 11 | I | 8.0 (5.3–10.6) | 2.6 (-0.1–5.2) | 0.059 | -0.8 (-4.7–3.0) | 0.667 |
| | 11 | C | 8.3 (5.5–11.1) | 3.4 (0.6–6.2) | 0.016 | | |
| Weakness | 0 | I | 10.5 (7.4–13.6) | NA | NA | NA | NA |
| | 0 | C | 11.0 (7.9–14.2) | NA | NA | NA | NA |
| | 2 | I | 12.8 (9.4–16.2) | 2.3 (-0.6–5.1) | 0.118 | -1.6 (-5.6–2.5) | 0.446 |
| | 2 | C | 14.9 (11.5–18.3) | 3.8 (0.9–6.7) | 0.009 | | |
| | 7 | I | 20.0 (17.2–22.9) | 9.5 (7.0–12.1) | <0.001 | -1.2 (-4.8–2.4) | 0.515 |
| | 7 | C | 21.8 (19.2–24.4) | 10.8 (8.1–13.4) | <0.001 | | |
| | 11 | I | 25.8 (23.1–28.6) | 15.4 (11.9–18.9) | <0.001 | -0.9 (-5.4–3.7) | 0.714 |
| | 11 | C | 27.3 (24.5–30.0) | 16.2 (12.5–19.9) | <0.001 | | |
| Weight loss | 0 | I | 9.0 (6.8–11.3) | NA | NA | NA | NA |
| | 0 | C | 7.2 (5.2–9.2) | NA | NA | NA | NA |
| | 2 | I | 8.3 (6.1–10.6) | -0.7 (-3.8–2.4) | 0.669 | -3.2 (-7.6–1.2) | 0.151 |
| | 2 | C | 9.7 (7.3–12.1) | 2.5 (-0.6–5.6) | 0.109 | | |
| | 7 | I | 8.8 (7.1–10.6) | -0.2 (-2.9–2.5) | 0.890 | -2.3 (-6.0–1.5) | 0.238 |
| | 7 | C | 9.2 (7.4–11.0) | 2.1 (-0.5–4.7) | 0.117 | | |
| | 11 | I | 9.2 (6.5–12.0) | 0.2 (-3.2–3.7) | 0.903 | -1.5 (-6.3–3.3) | 0.535 |
| | 11 | C | 8.9 (6.2–11.6) | 1.7 (-1.6–5.0) | 0.306 | | |
| Slowness | 0 | I | 0.8 (0.2–1.3) | NA | NA | NA | NA |
| | 0 | C | 1.3 (0.4–2.2) | NA | NA | NA | NA |
| | 2 | I | 1.1 (0.4–1.9) | 0.4 (-0.3–1.1) | 0.267 | -0.2 (-1.4–1.0) | 0.722 |
| | 2 | C | 1.9 (0.9–3.0) | 0.6 (-0.4–1.6) | 0.216 | | |
| | 7 | I | 3.6 (2.3–5.0) | 2.9 (1.7–4.1) | <0.001 | 0.2 (-1.4–1.9) | 0.775 |
| | 7 | C | 3.9 (2.5–5.3) | 2.6 (1.4–3.9) | <0.001 | | |
| | 11 | I | 8.4 (5.2–11.7) | 7.7 (4.5–10.8) | <0.001 | 2.1 (-2.0–6.2) | 0.314 |

| | | | | |
|---|---|---|---|---|
| 11 | C | 6.9 (4.0–9.8) | 5.6 (2.7–8.4) | <0.001 |

The best-fitting mixed-effects logistic regression model, where time had a non-linear effect and was split into two linear periods (t2=0–2 years, t11=2–11 years) was used to estimate the prevalence of each frailty component (%), the change from baseline (percentage points), and the difference in this change (percentage points) between intervention and control groups (time x randomization group interaction). Baseline age, sex, protein intake (g/kg), number of chronic diseases (0, 1, 2, ≥3), and education (years) were used as covariates. In the difference between groups, a negative value indicates that the difference is in favor of the intervention group.

*Abbreviations: CI=confidence interval, I=intervention, C=control.*
